# Supplementary material for: Toward Efficient and Accurate EMRI Parameter Estimation: A Machine Learning-Enhanced MCMC Framework
Source: Research (Wash D C). 2026 Jan 8;9:1055. doi: 10.34133/research.1055 (PMC13229011; doi:10.34133/research.1055)
Supplement: Supplementary 1 — Notes S1 to S4 Figs. S1 to S6 [file research.1055.f1.zip › Supplemental Material.pdf]

Supplementary Materials for Towards Efficient and Accurate EMRI Parameter Estimation: A Machine Learning-Enhanced MCMC Framework

Bo Liang[1†,2], Chang Liu[1†,2,3], Hanlin Song[4], Zhenwei Lyu[5], Minghui Du[1*], Peng Xu[1*,2,6], Ziren Luo[1,2,7], Sensen He[8], Haohao Gu[8], Tianyu Zhao[1], Manjia Liang[1], Yuxiang Xu[1], Li-e Qiang[3], Mingming Sun[9], and Wei-Liang Qian[10]

[1]Center for Gravitational Wave Experiment, National Microgravity Laboratory, Institute of Mechanics, Chinese Academy of Sciences, Beijing 100190, China

[2]Taiji Laboratory for Gravitational Wave Universe (Beijing/Hangzhou), University of Chinese Academy of Sciences (UCAS), Beijing 100049, China

[3]National Space Science Center, Chinese Academy of Sciences, Beijing 100190, China

[4]School of Physics, Peking University, Beijing 100871, China

[5]Leicester International Institute, Dalian University of Technology, Panjin 124221, China

[6]Lanzhou Center of Theoretical Physics, Lanzhou University, Lanzhou 730000, China

[7]Key Laboratory of Gravitational Wave Precision Measurement of Zhejiang Province, Hangzhou Institute for Advanced Study, UCAS, Hangzhou 310024, China

[8]Baidu Inc., Beijing 100085, P. R. China

[9]AGI Lab, Beijing Institute of Mathematical Sciences and Applications, Beijing, China

[10]Escola de Engenharia de Lorena, Universidade de São Paulo, 12602-810, Lorena, SP, Brazil

[*]duminghui@imech.ac.cn, xupeng@imech.ac.cn

[†]These authors contributed equally to this work.

Note S1 FM-MCMC Walk Results

In this note, we provide additional walk results for the FM-MCMC framework, showing the posterior distributions for walkers 0 and 7. As shown in Fig. S1, these walkers clearly demonstrate the multimodal nature of the posterior distribution, revealing how FM-MCMC is able to explore multiple modes effectively. The walkers successfully navigate between different modes of the likelihood surface, providing a clear illustration of FM-MCMC's ability to avoid local maxima and explore the global posterior distribution.

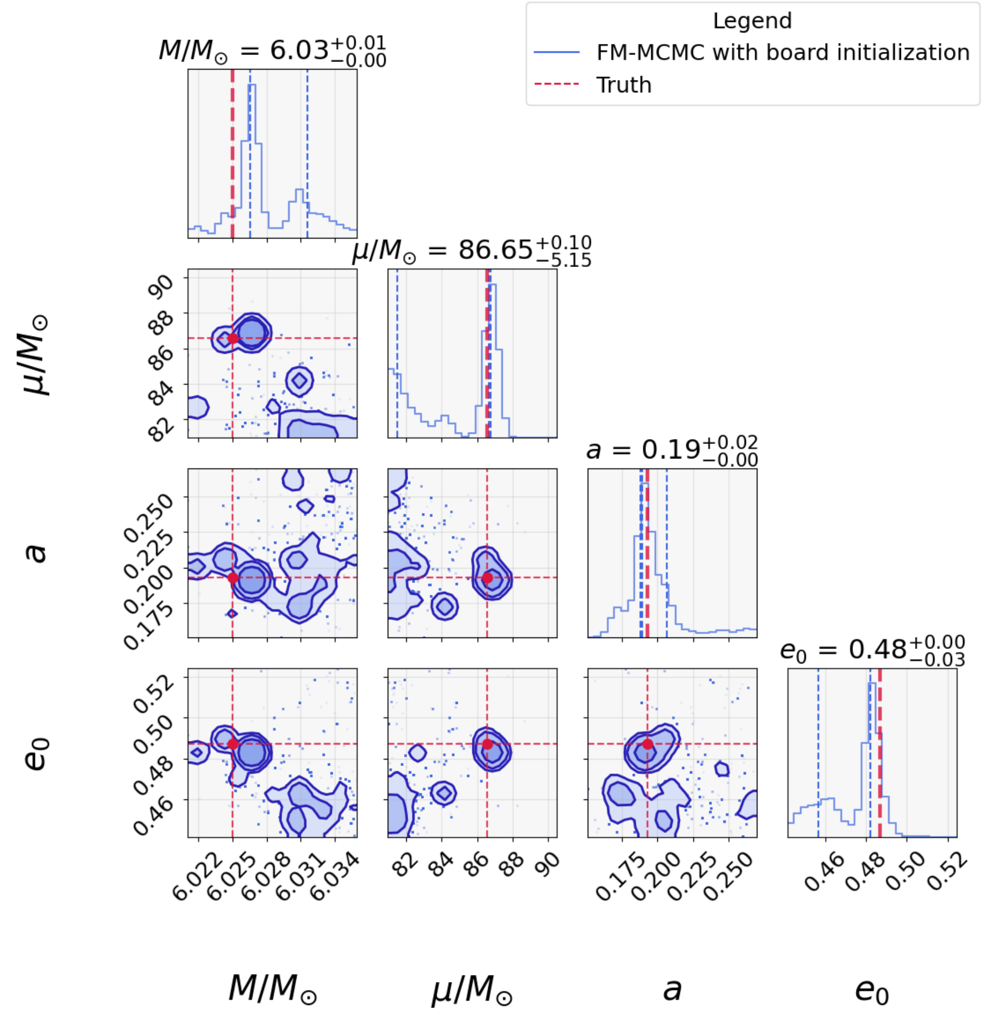

Fig. S1: This figure shows the posterior distribution for walkers 0 and 7 in the FM-MCMC run. The plot highlights the multimodal structure of the posterior distribution, with clear evidence that FM-MCMC is able to explore multiple modes of the likelihood surface. The walkers effectively navigate between different modes, demonstrating the method's superior global exploration capabilities.

In this note, we also present additional walk results for both FM-MCMC and PT-MCMC methods to demonstrate the multimodal nature of the posterior distribution more clearly. As shown in Fig. S2

and Fig. S3, the posterior distributions exhibit multiple modes, which are explored more effectively by FM-MCMC compared to PT-MCMC.

Note S2 Comparison with Neural Posterior Estimation

In addition to the main comparisons with Eryn, we further evaluate our FMPE framework against Neural Posterior Estimation (NPE), which represents another widely used class of neural posterior estimation techniques. To ensure fairness, both FMPE and NPE were implemented with the same base neural architecture. However, a crucial distinction is that NPE requires neural spline flows for density estimation. In our setup, we employed six neural spline flows, which significantly increases the number of trainable parameters in the model. As a result, the model size of NPE is approximately 300 million parameters, while FMPE contains only about 100 million parameters. In terms of training cost, NPE requires around three days of training on a single NVIDIA RTX 4090 GPU, whereas FMPE converges within about 20 hours under the same hardware conditions. The calibration performance is further demonstrated by the P–P plots in Figure 5 (FMPE) and Fig. S4 (NPE). While both methods yield statistically valid posterior samples, the average p-values across all intrinsic parameters are consistently higher for FMPE than for NPE, indicating better overall calibration.

Note S3 Results under Fully Uninformed Initialization

In this note, we present the results of EMRI parameter estimation when all eight parameters, including both intrinsic parameters (M, μ, a, e_0) and extrinsic parameters (θ_S, ϕ_S, θ_K, ϕ_K), are initialized under fully uninformed priors. Specifically, the MCMC chains are initialized by random sampling across the entire prior ranges as defined in Table 1, without any constraints near the injected true values. This setup provides a stringent test of the robustness of our method, as it reflects the most challenging scenario for realistic searches in which no prior information is assumed. To ensure a fair comparison, both FM-MCMC and PT-MCMC were run with identical parallel tempering configurations: 20 walkers and 20 temperature ladder levels. In Fig. S5 We include detailed convergence diagnostics for both FM-MCMC and PT-MCMC, showing walker traces for all eight parameters. In addition, we present the posterior distributions of the intrinsic parameters under this setting in Fig. S6. The results clearly demonstrate that FM-MCMC achieves robust convergence to the true posterior modes, even when initialized from completely uninformed states. By contrast, PT-MCMC fails to consistently escape local optima, leading to biased or unconverged results. These findings confirm the robustness of FM-MCMC under fully uninformed initialization and further validate the conclusions drawn in the main text. We note that while FM-MCMC is capable of handling intrinsic parameters effectively in this setting, accurate recovery of extrinsic parameters may require further developments. In particular, more advanced neural network architectures, fine-tuning strategies, or hierarchical inference approaches may be necessary to improve convergence for spin polar angle parameters. Exploring these directions will be an important focus of future work.

Note S4 Network Implementation

The CNF is trained on a dataset of 20,000 simulated EMRI waveforms. During training, independent Gaussian noise realizations are dynamically added to each clean signal at every epoch, ensuring that the model learns to generalize across varying noise conditions rather than overfitting to specific noise samples. Each waveform corresponds to a two-month Taiji observation sampled at 0.1 Hz, yielding a time series of approximately 200,000 data points per channel.

The feature extraction network preceding the flow model is implemented as a convolutional neural network specifically designed to handle long-duration gravitational-wave time series. The network begins with a large-kernel convolutional layer (kernel size 65, stride 32) that performs strong temporal downsampling while preserving key waveform features. It is followed by a stack of six residual blocks with dilation factors of $1, 2, 4, 8, 16, 32$, enabling the model to capture both short- and long-timescale dependencies effectively. Each residual block consists of two 1D convolutional layers (kernel size 3, dilation d) with identical channel dimensions, followed by Batch Normalization and GELU activation. The residual connection directly adds the block input to the output of the second convolution, ensuring stable gradient flow and preserving information across layers. The final GELU activation provides smooth nonlinear transformation, enhancing the model's expressivity for complex waveform structures. After the residual stack, a sequence of convolution, pooling, and fully connected projection layers further compresses the representation into a 2048-dimensional latent embedding.

The second core component is the deep conditional normalizing flow network itself, which is structured as a sequence of 21 residual blocks. This network is designed to perform a progressive, hierarchical transformation of features. It begins by processing a combined input of the compressed data representation, the latent parameters, and an embedded time variable, with an initial dimension of 3072. Through its deep structure, it systematically refines and compresses this information across multiple layers, ultimately mapping it down to the 4-dimensional vector field that defines the probability flow. The architecture employs a symmetric, funnel-like design, starting with wider layers (e.g., 8192 units) to capture broad features and gradually narrowing through successive blocks (e.g., 4096, 2048, 1024 units and so forth) to distill increasingly fine-grained information, culminating in the final output dimension.

This hierarchical design facilitates a synergistic optimization between the compressed signal embedding, the flow time parameter, and the dynamic state of the source parameters. The training was conducted on a single NVIDIA RTX 4090 GPU using the Adam optimizer with an initial learning rate of 1e-4, which was decayed following a cosine annealing schedule over the course of 2000 epochs. The complete training process required approximately 12 hours to complete.

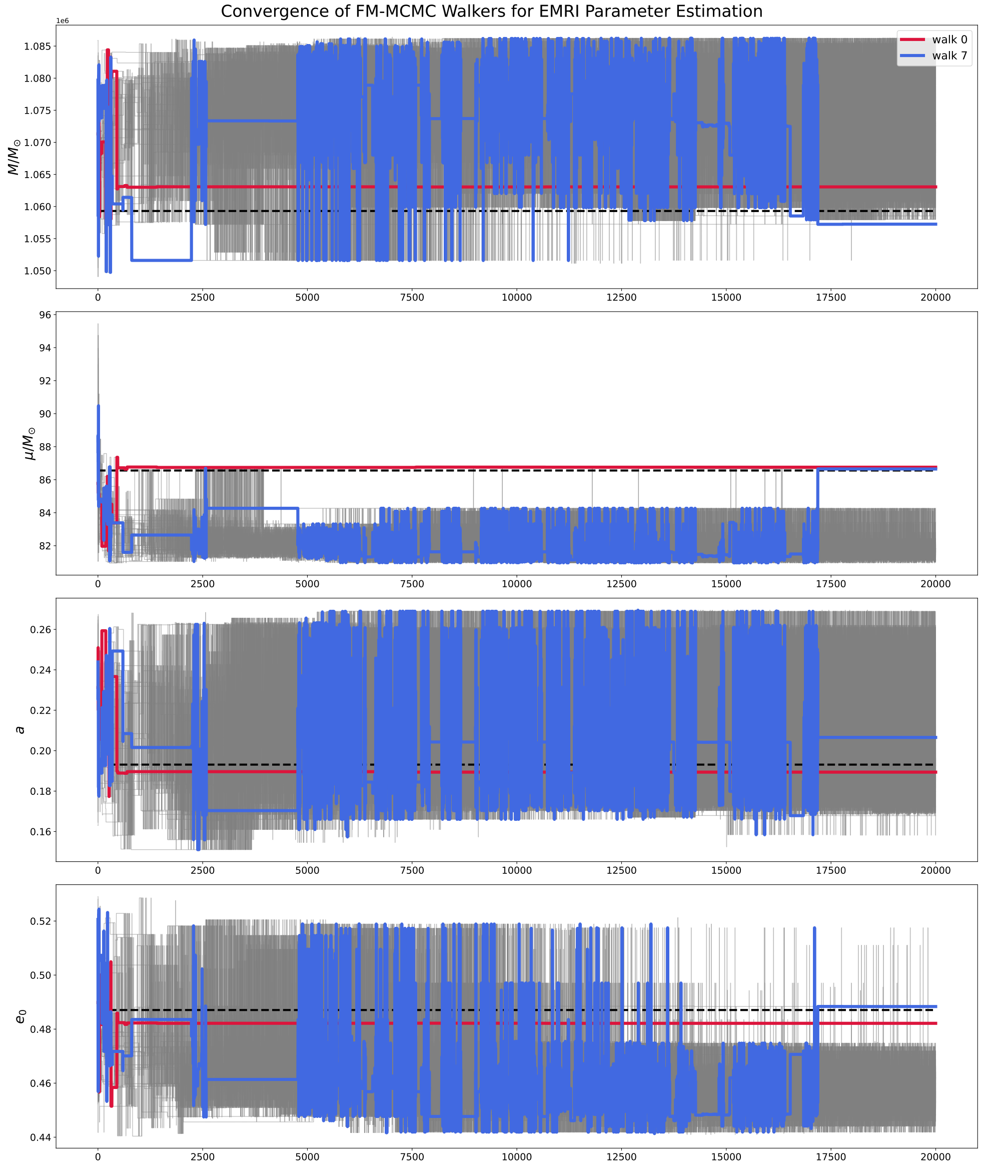

Fig. S2: This figure shows the walk results for FM-MCMC on the posterior distribution of the EMRI parameters. The plot demonstrates the multimodal nature of the posterior surface, where FM-MCMC successfully explores multiple modes of the likelihood. The walkers effectively navigate through global and local optima, highlighting FM-MCMC's ability to explore the full parameter space.

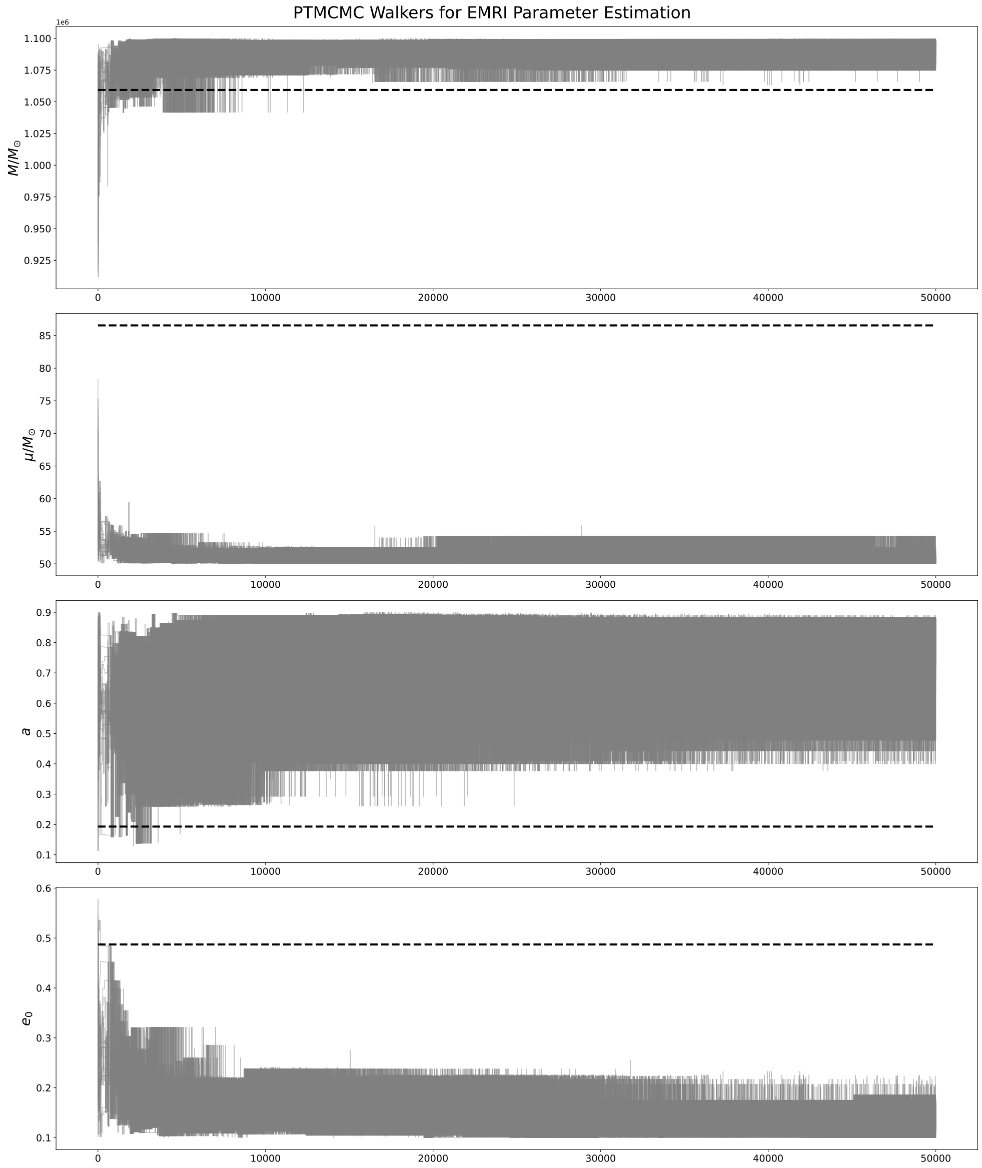

Fig. S3: This figure shows the walk results for PT-MCMC on the posterior distribution of the EMRI parameters. The plot illustrates how PT-MCMC gets trapped in local maxima and fails to explore other regions of the posterior distribution. Despite the use of parallel tempering, PT-MCMC is limited in its ability to explore multiple modes in the posterior.

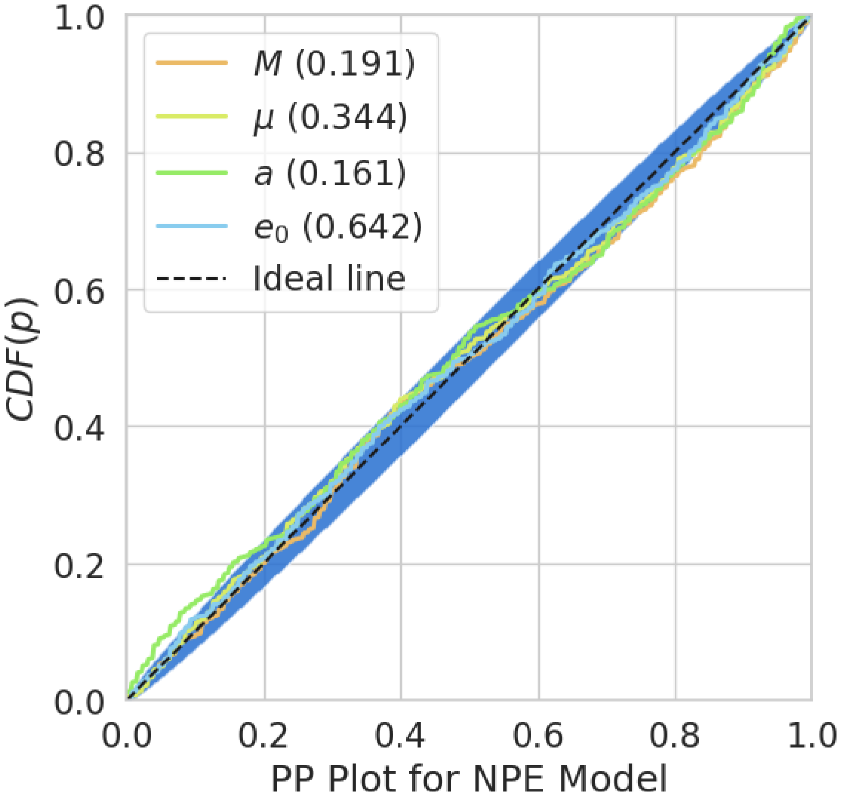

Fig. S4: PP plot for NPE model, based on 500 simulated EMRI signals. The dashed linerepresents the ideal case, while the colored lines represent the empirical CDFs of the parameters. The shaded blue region indicates the 95% confidence bands.

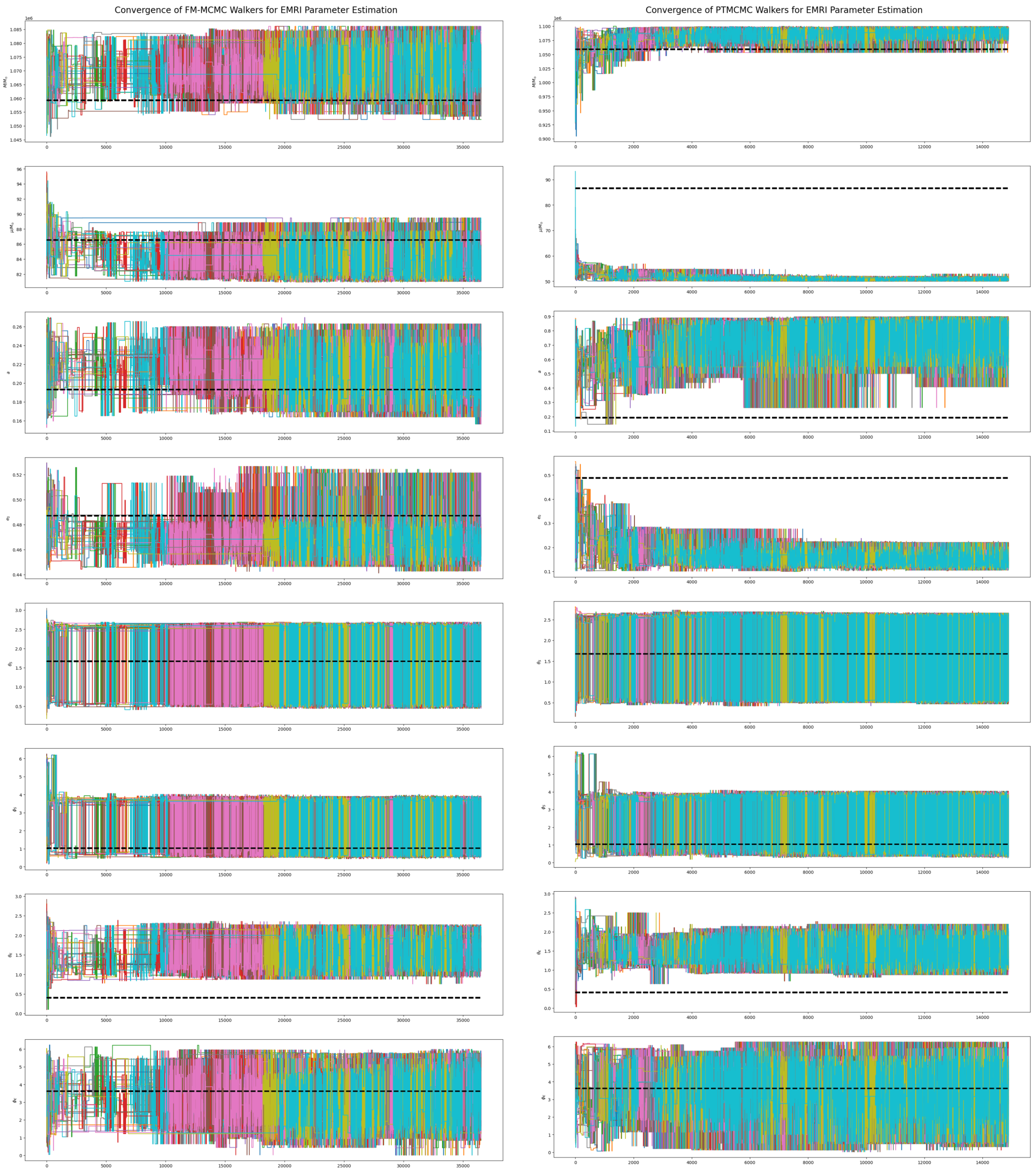

Fig. S5: **FM-MCMC vs. PT-MCMC under fully uninformed initialization.** Convergence diagnostics and posterior distributions under fully uninformed initialization. All eight parameters, including both intrinsic parameters (M, μ, a, e_0) and extrinsic parameters (θ_S, ϕ_S, θ_K, ϕ_K), are initialized by random sampling across their full prior ranges as defined in Table 1. (Left) For FM-MCMC, the walker traces show rapid convergence toward the true posterior modes, and the corresponding posterior distributions of the intrinsic parameters accurately recover the injected values. (Right) For PT-MCMC, the chains remain trapped in local optima, and the posterior distributions exhibit significant bias, demonstrating its difficulty in exploring the multimodal landscape. These results validate the robustness of FM-MCMC under the most challenging initialization scenario, while highlighting the limitations of PT-MCMC in fully uninformed searches.

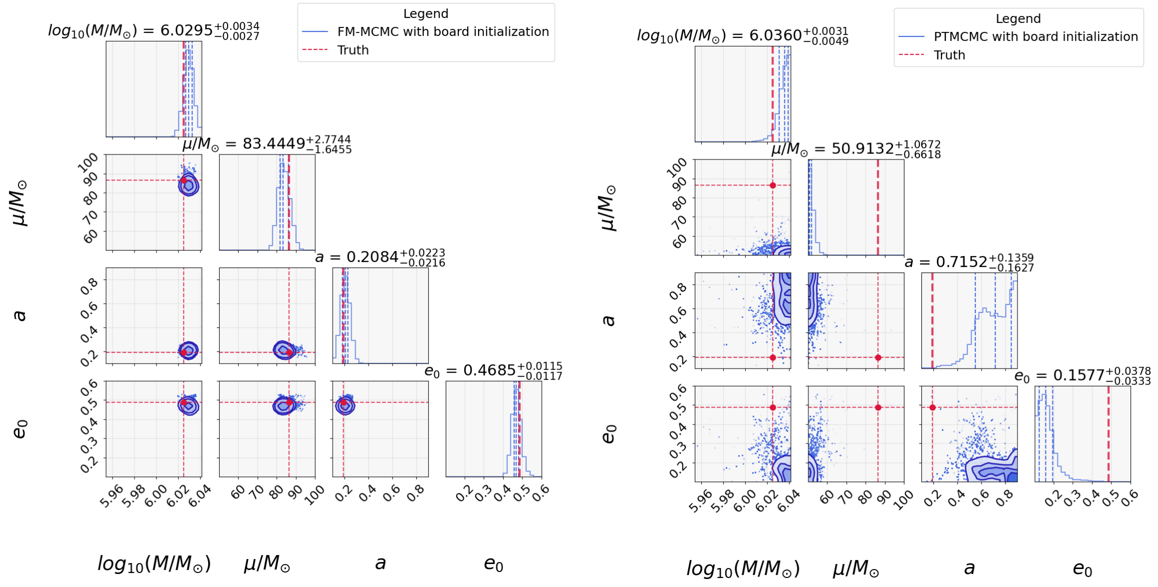

Fig. S6: **Comparison of posterior distributions under fully uninformed initialization.** (Left) FM-MCMC successfully recovers the injected values of the intrinsic parameters (M, μ, a, e_0) with tight posterior distributions, demonstrating robust convergence to the true global modes. (Right) PTMCMC results show significant bias and broader distributions, indicating that the sampler remains trapped in local optima. These results highlight the superior ability of FM-MCMC to explore the multimodal likelihood surface compared to PTMCMC.
