# Supplementary figures and images for: Toward Efficient and Accurate EMRI Parameter Estimation: A Machine Learning-Enhanced MCMC Framework
Source: Research (Wash D C). 2026 Jan 8;9:1055. doi: 10.34133/research.1055 (PMC13229011; doi:10.34133/research.1055)

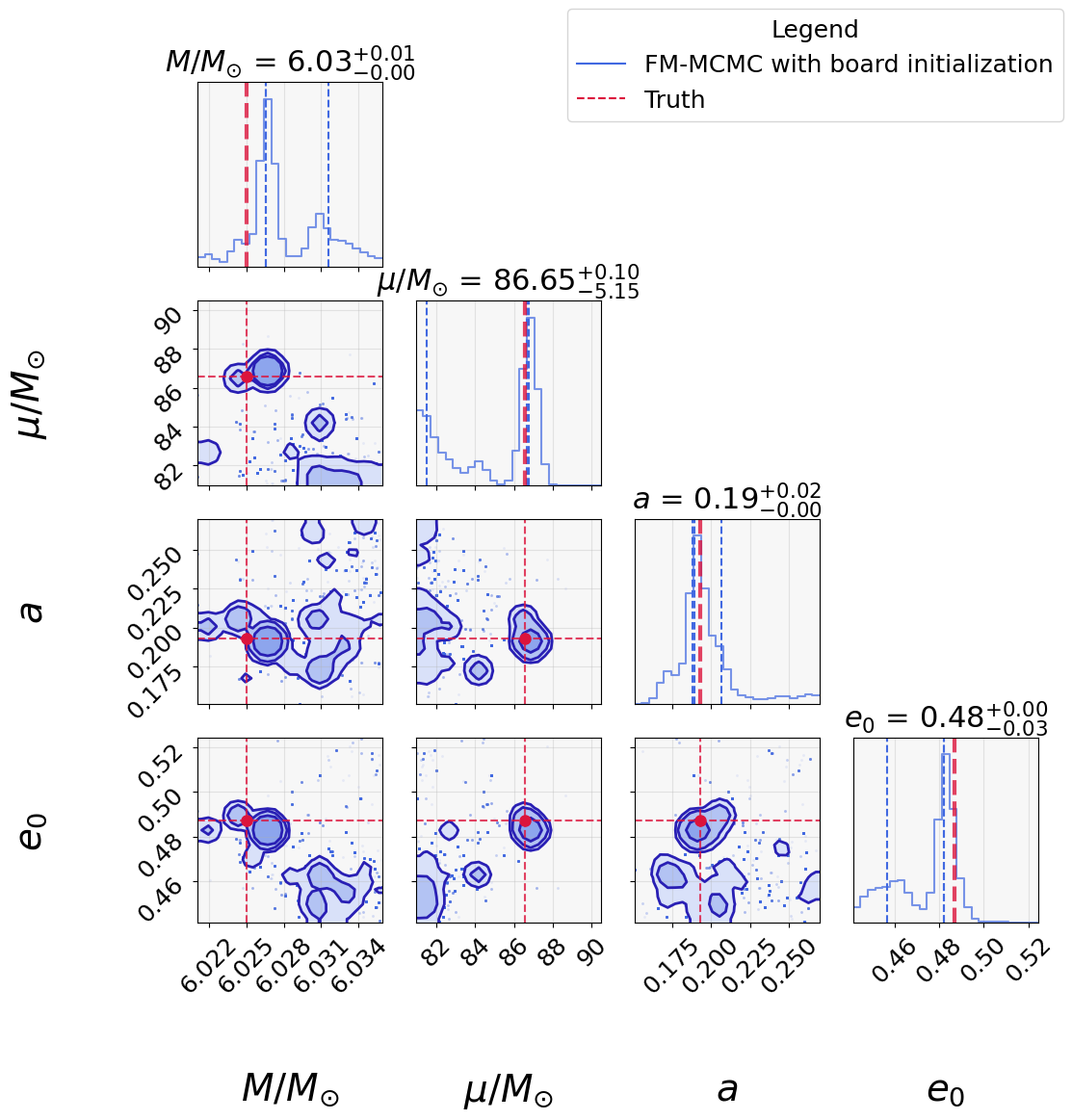

Supplement: Supplementary 1 — Notes S1 to S4 Figs. S1 to S6 [file research.1055.f1.zip › Figure S1.png]

# Convergence of FM-MCMC Walkers for EMRI Parameter Estimation

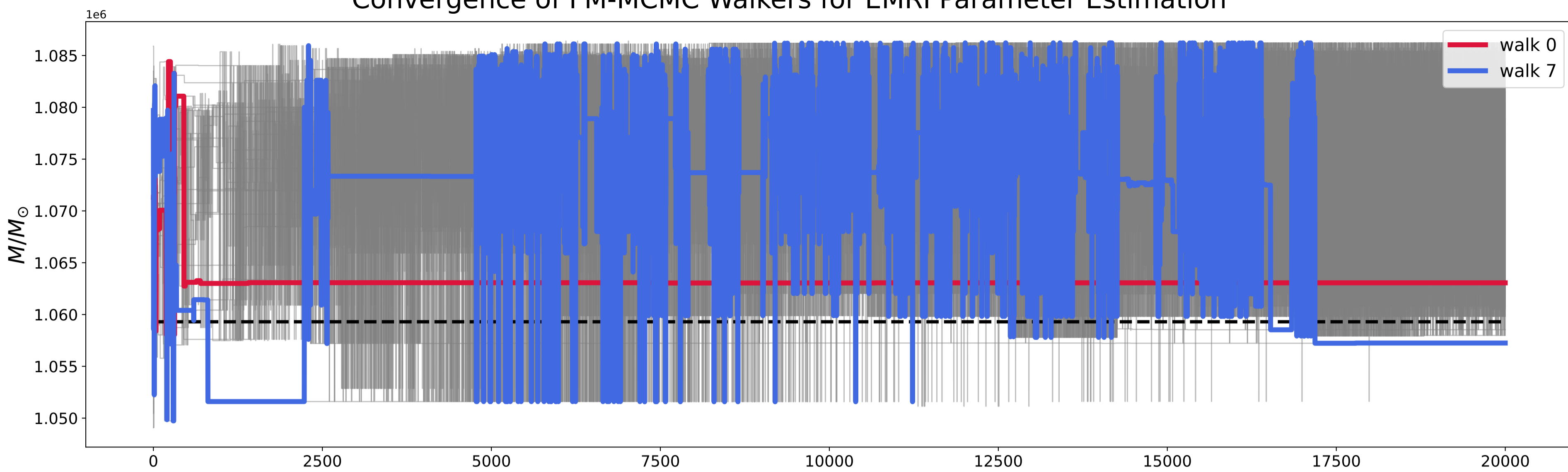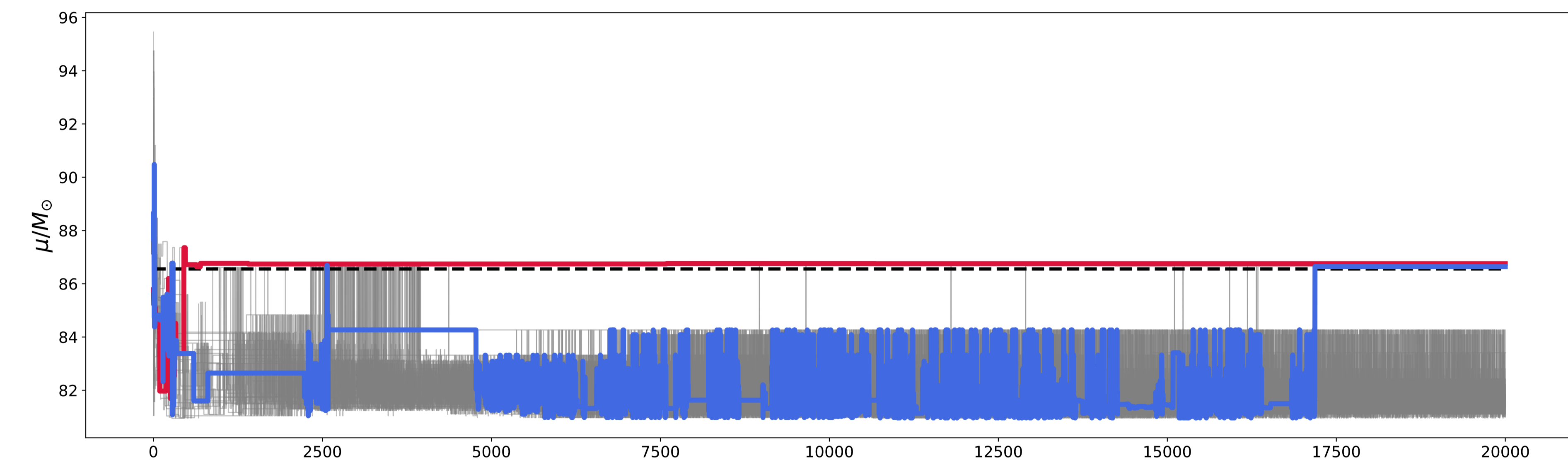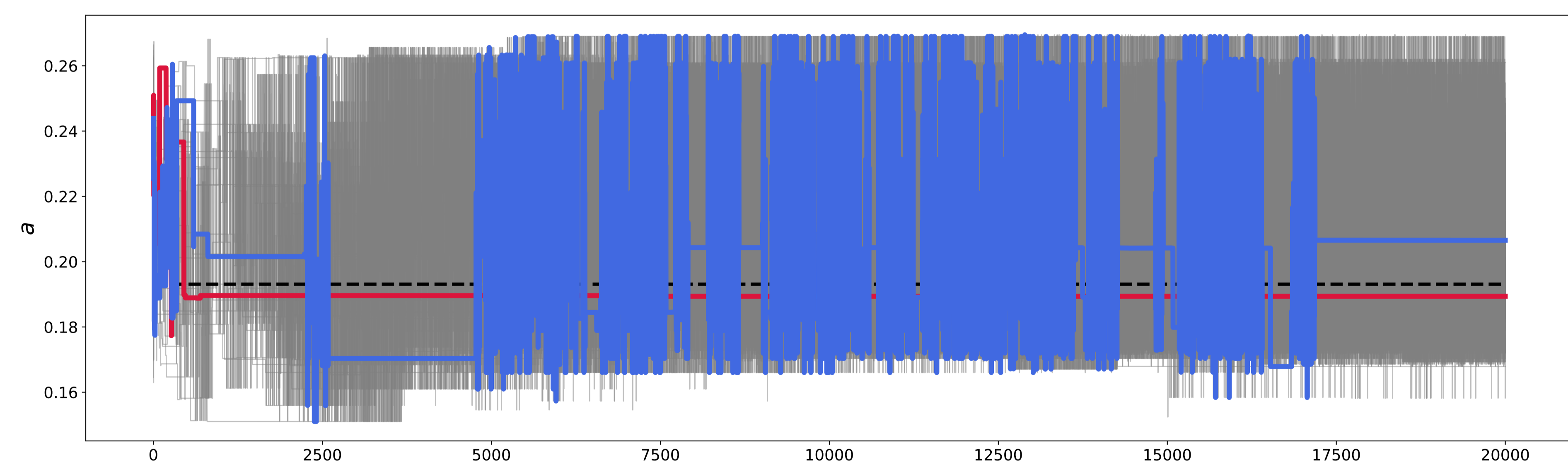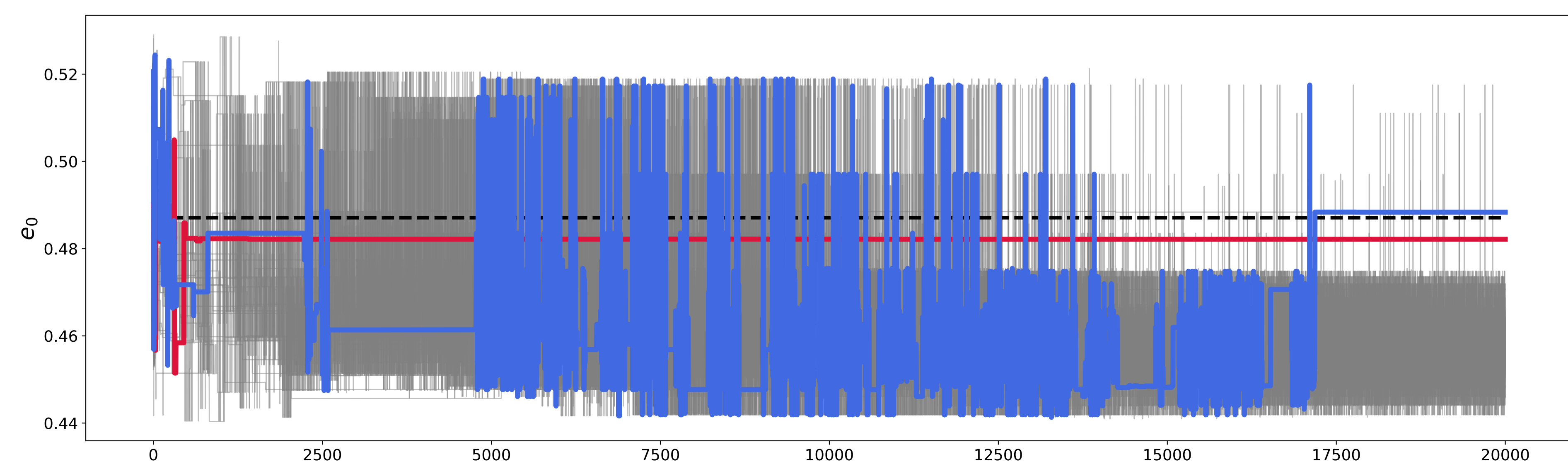

Supplement: Supplementary 1 — Notes S1 to S4 Figs. S1 to S6 [file research.1055.f1.zip › Figure S2.pdf]

PTMCMC Walkers for EMRIParameter Estimation

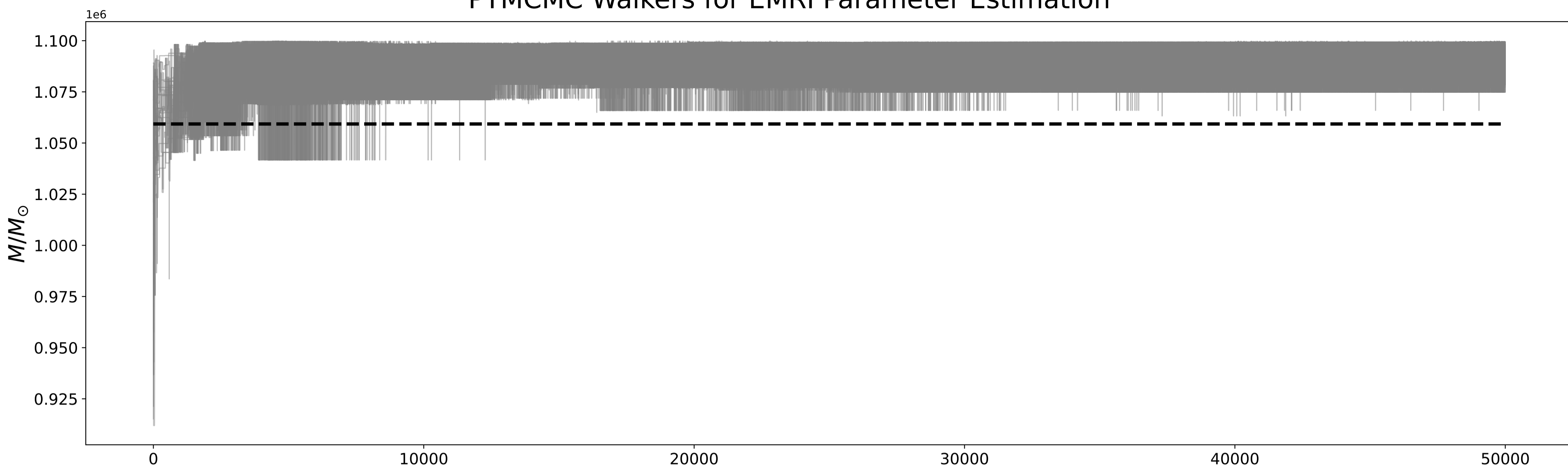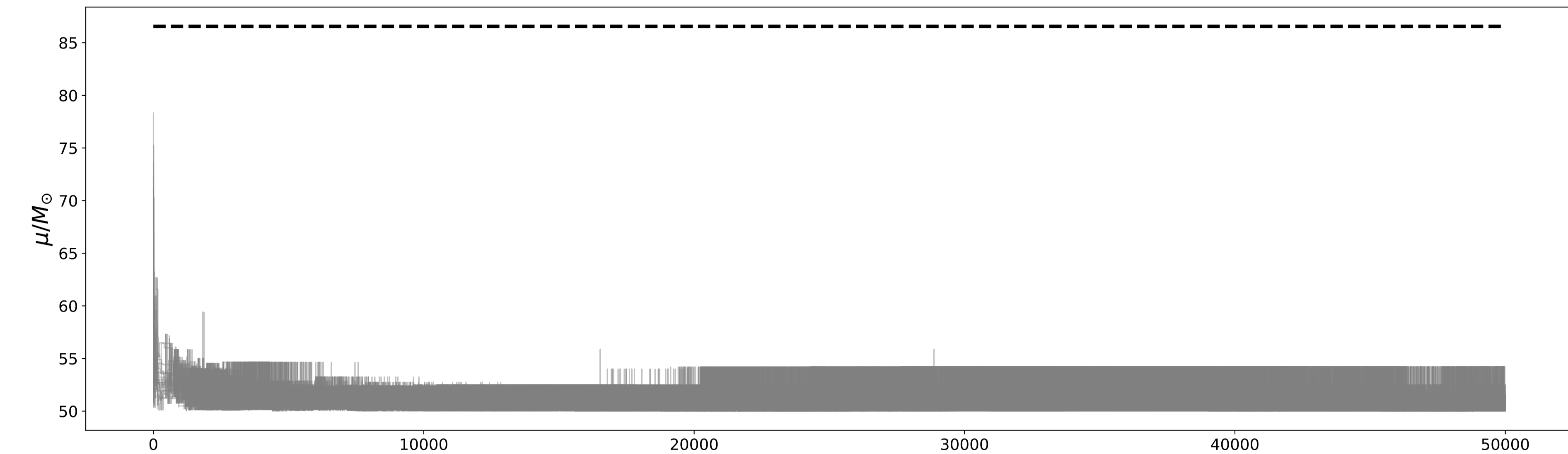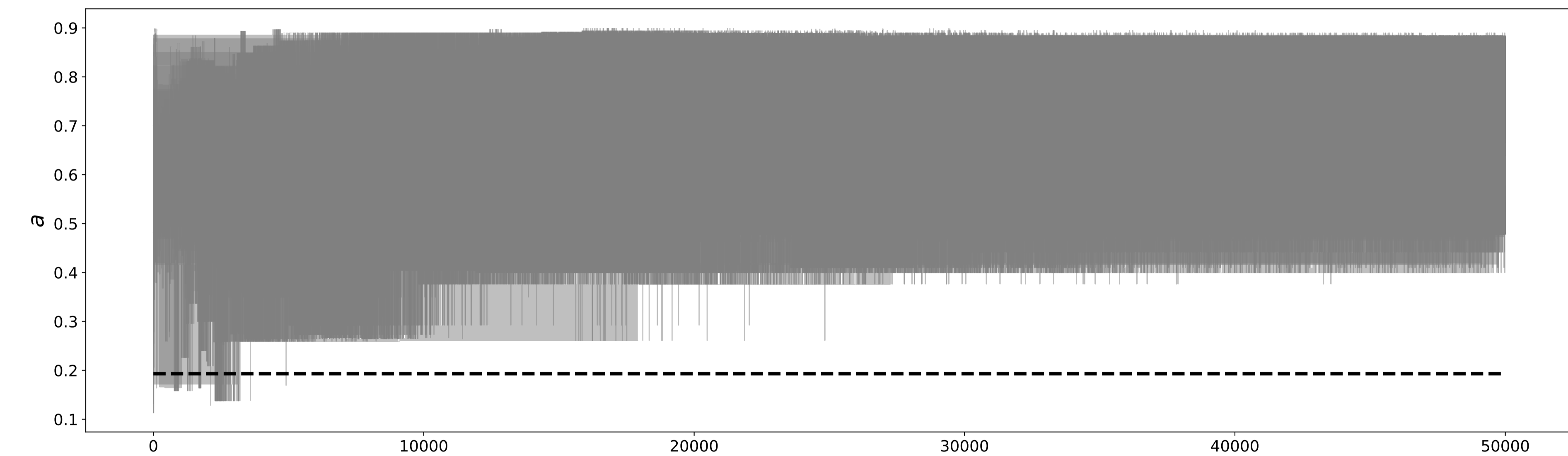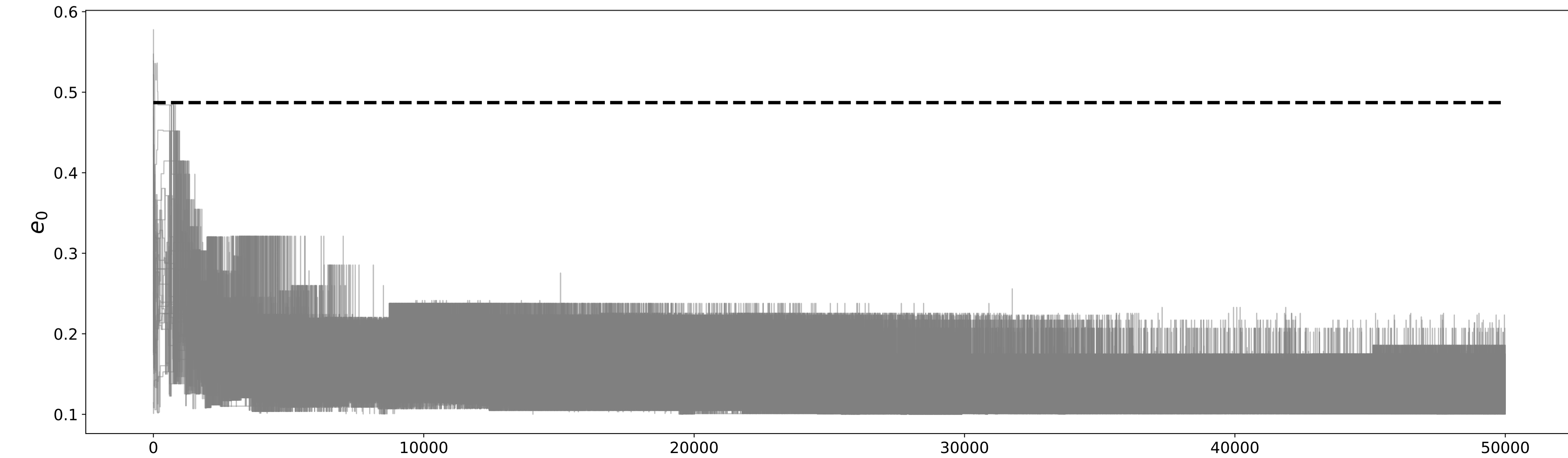

Supplement: Supplementary 1 — Notes S1 to S4 Figs. S1 to S6 [file research.1055.f1.zip › Figure S3.pdf]

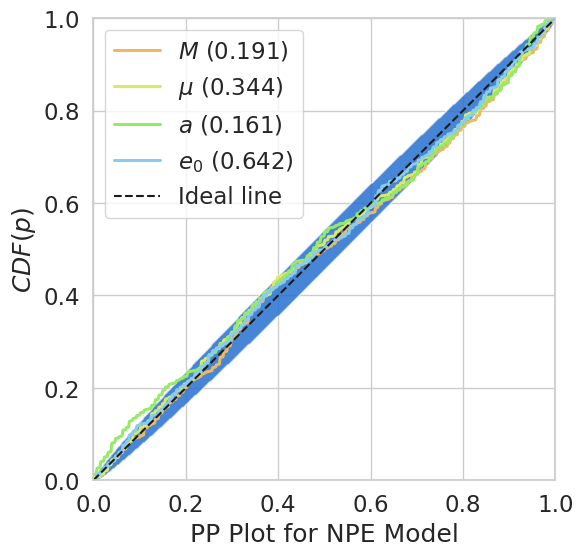

Supplement: Supplementary 1 — Notes S1 to S4 Figs. S1 to S6 [file research.1055.f1.zip › Figure S4.png]

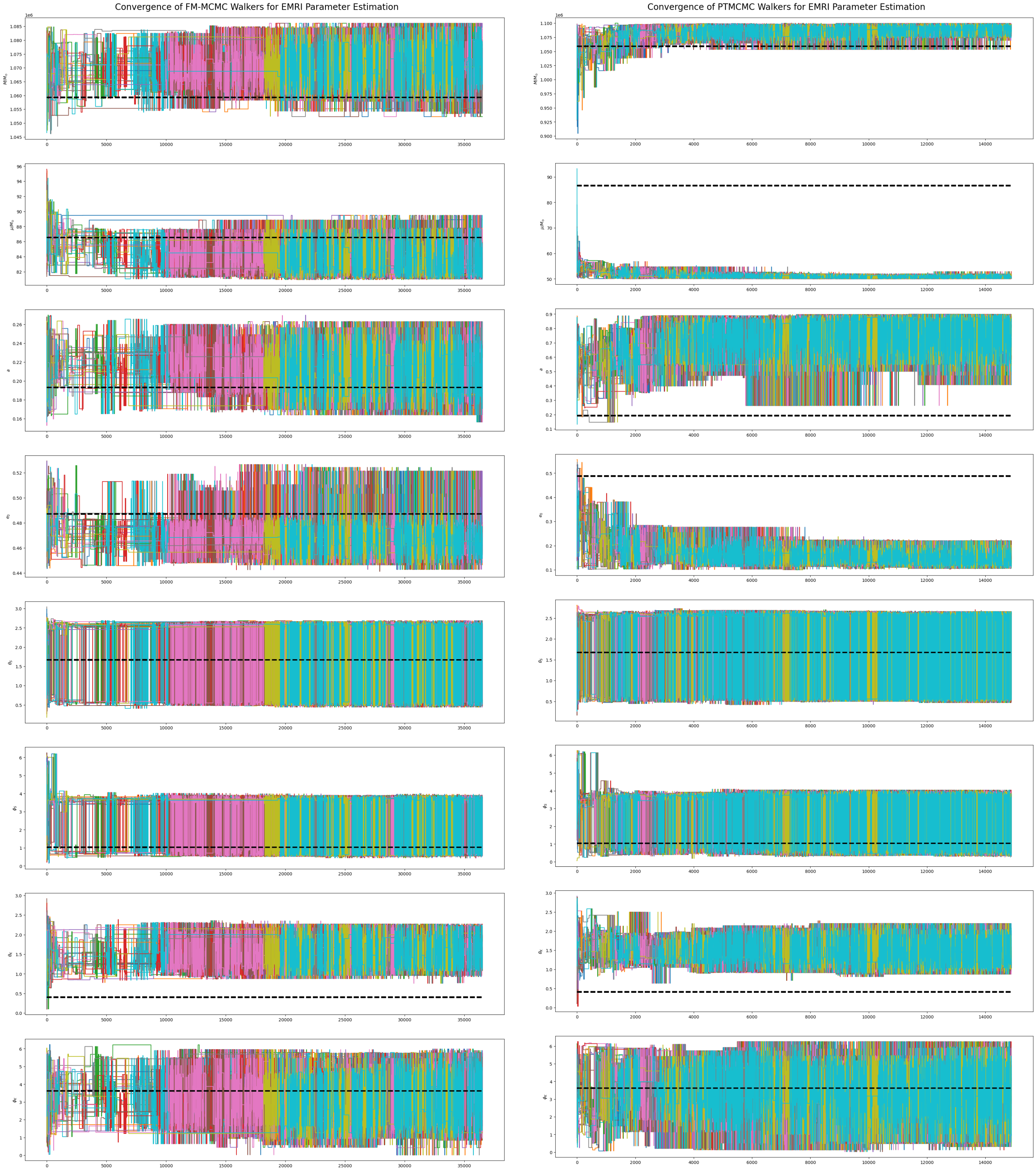

Supplement: Supplementary 1 — Notes S1 to S4 Figs. S1 to S6 [file research.1055.f1.zip › Figure S5.png]

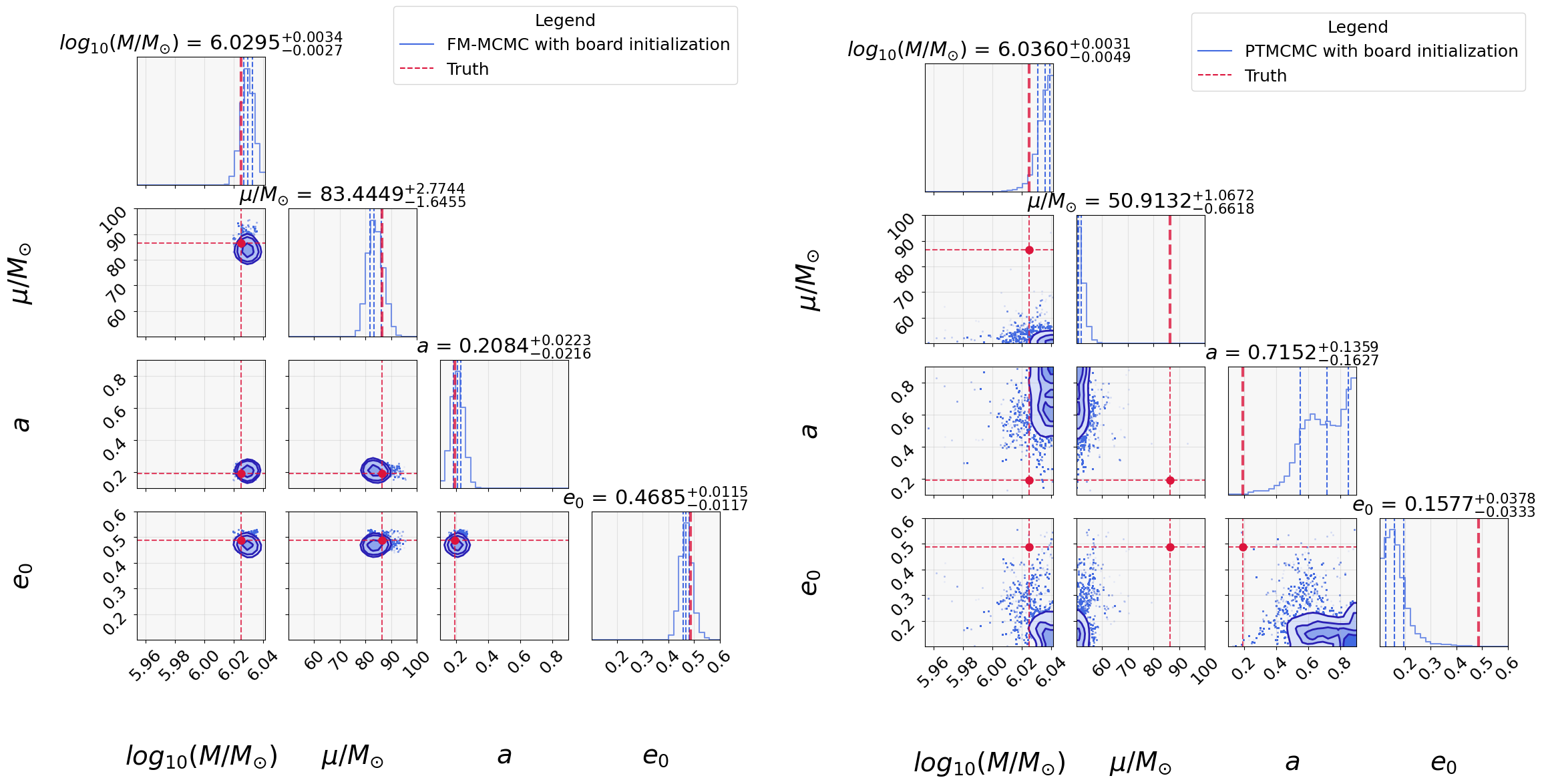

Supplement: Supplementary 1 — Notes S1 to S4 Figs. S1 to S6 [file research.1055.f1.zip › Figure S6.png]
